# A Novel DNA Methylation-Based Signature Can Predict the Responses of MGMT Promoter Unmethylated Glioblastomas to Temozolomide

**DOI:** 10.3389/fgene.2019.00910

**Published:** 2019-09-27

**Authors:** Rui-Chao Chai, Yu-Zhou Chang, Qiang-Wei Wang, Ke-Nan Zhang, Jing-Jun Li, Hua Huang, Fan Wu, Yu-Qing Liu, Yong-Zhi Wang

**Affiliations:** ^1^Department of Molecular Neuropathology, Beijing Neurosurgical Institute, Beijing Tiantan Hospital, Capital Medical University, Beijing, China; ^2^China National Clinical Research Center for Neurological Diseases, Beijing Tiantan Hospital, Capital Medical University, Beijing, China; ^3^Department of Neurosurgery, Beijing Tiantan Hospital, Capital Medical University, Beijing, China

**Keywords:** glioblastoma, DNA methylation, temozolomide, MGMT, signature

## Abstract

Glioblastoma (GBM) is the most malignant glioma, with a median overall survival (OS) of 14–16 months. Temozolomide (TMZ) is the first-line chemotherapy drug for glioma, but whether TMZ should be withheld from patients with GBMs that lack O6-methylguanine-DNA methyltransferase (*MGMT*) promoter methylation is still under debate. DNA methylation profiling holds great promise for further stratifying the responses of *MGMT* promoter unmethylated GBMs to TMZ. In this study, we studied 147 TMZ-treated *MGMT* promoter unmethylated GBM, whose methylation information was obtained from the HumanMethylation27 (HM-27K) BeadChips (n = 107) and the HumanMethylation450 (HM-450K) BeadChips (n = 40) for training and validation, respectively. In the training set, we performed univariate Cox regression and identified that 3,565 CpGs were significantly associated with the OS of the TMZ-treated *MGMT* promoter unmethylated GBMs. Functional analysis indicated that the genes corresponding to these CpGs were enriched in the biological processes or pathways of mitochondrial translation, cell cycle, and DNA repair. Based on these CpGs, we developed a 31-CpGs methylation signature utilizing the least absolute shrinkage and selection operator (LASSO) Cox regression algorithm. In both training and validation datasets, the signature identified the TMZ-sensitive GBMs in the *MGMT* promoter unmethylated GBMs, and only the patients in the low-risk group appear to benefit from the TMZ treatment. Furthermore, these identified TMZ-sensitive *MGMT* promoter unmethylated GBMs have a similar OS when compared with the *MGMT* promoter methylated GBMs after TMZ treatment in both two datasets. Multivariate Cox regression demonstrated the independent prognostic value of the signature in TMZ-treated *MGMT* promoter unmethylated GBMs. Moreover, we also noticed that the hallmark of epithelial–mesenchymal transition, ECM related biological processes and pathways were highly enriched in the MGMT unmethylated GBMs with the high-risk score, indicating that enhanced ECM activities could be involved in the TMZ-resistance of GBM. In conclusion, our findings promote our understanding of the roles of DNA methylation in *MGMT* umethylated GBMs and offer a very promising TMZ-sensitivity predictive signature for these GBMs that could be tested prospectively.

## Introduction

Glioma is the most common type of malignant brain tumor in adults (Jiang et al., 2016; Chai et al., 2019b). Glioblastoma (GBM, WHO IV) is the most malignant glioma, accounting for 50–60% of total glioma (Louis et al., 2016). Currently, the prognosis for patients with GBM is still dismal, with a median overall survival (OS) of 14–16 months (Jiang et al., 2016; Louis et al., 2016; Chai et al., 2019a). The alkylating agent temozolomide (TMZ) is the first-line chemotherapy drug for glioma. TMZ is used concurrently with radiation and then provided as monotherapy during adjuvant treatment. The promoter methylation level of the O6-methylguanine-DNA methyltransferase (*MGMT*), a ubiquitous DNA repair enzyme which can rapidly reverse alkylation at the O6 position, has been acknowledged as a predictive marker for TMZ sensitivity (Hegi et al., 2005; Chai et al., 2019a; Chai et al., 2019e). *MGMT* promoter methylated GBM displays higher sensitivity to TMZ treatment than *MGMT* promoter unmethylated GBM (Hegi et al., 2005; Wick et al., 2014; Chai et al., 2019a). However, we noticed that the prognosis for TMZ treated *MGMT* promoter unmethylated GBM is still largely heterogeneous, indicating that some other factors may also affect the sensitivity of *MGMT* promoter unmethylated GBM to TMZ treatment. Thus, further stratification of these GBM is urgently needed.

In the central nervous system, DNA methylation profiling has been used as a robust and reproducible method to further stratify the tumors into different subgroups (Sturm et al., 2012; Pajtler et al., 2015; Sturm et al., 2016). Moreover, general DNA methylation or a group of CpGs methylation profiling could also serve as biomarkers to evaluate drug- or radio-therapeutic sensitivity in various diseases, including tumors (Kumar et al., 2018; Zhao et al., 2018b; Chen et al., 2019b). In a recent study, a five-CpG DNA methylation score has shown its value in predicting metastatic-lethal outcomes of males suffering localized prostate cancer, treated with radical prostatectomy (Zhao et al., 2018b). The rapid accumulation of DNA methylation datasets makes it also possible to further stratify the glioma and may uncover novel biomarkers for management of gliomas. Recently, DNA methylation profiling of 23 DNA damage response (DDR) genes was shown to be associated with benefit from RT or TMZ therapy in IDH mutant low-grade glioma (Bady et al., 2018). Nevertheless, whether a group of CpGs DNA methylation profiling can predict the TMZ sensitivity of *MGMT* promoter unmethylated GBM remains unclear.

Here, we aimed to identify TMZ-sensitive GBMs in the entity of *MGMT* promoter unmethylated GBMs, using DNA methylation profiling. We adopted 107 and 40 TMZ treated *MGMT* promoter unmethylated GBMs as the training set and the validation set, respectively. We identified a list of CpGs whose methylation levels are significantly associated with the OS of TMZ-treated MGMT promoter unmethylated GBMs by univariate Cox regression analyses. Based on this, we developed a 31-CpGs TMZ therapeutic prognosis risk signature in the *MGMT* promoter unmethylated GBMs. This risk signature could successfully identify a subgroup of TMZ treated *MGMT* promoter unmethylated GBMs which have a similar prognosis when compared with the TMZ treated *MGMT* promoter methylated GBMs.

## Materials and Methods

### Samples Information

A total of 376 cases were enrolled in this study according to the following criteria: (a) diagnosed with GBM; (b) the DNA methylation data could be obtained; (c) the TMZ treatment option is available. The DNA methylation data and corresponding clinicopathological features for these cases were obtained from The Cancer Genome Atlas (TCGA) (http://cancergenome.nih.gov/). Within the 376 cases, the DNA methylation information of 279 cases (the 27K cohort) was collected from the HumanMethylation27 (HM-27K) BeadChips dataset, and the other 97 cases (the 450K cohort) were obtained from the HumanMethylation450 (HM-450K) BeadChips dataset. Clinicopathological information for all cases is summarized in **Supplementary Table 1**.

Of all 279 cases in the 27K cohort, 107 cases who received TMZ treatment and also with unmethylated *MGMT* were used to investigate the TMZ therapeutic prognosis value of CpGs methylation levels, and we also developed a risk signature using these cases. Of the 97 cases in the 450K cohort, 40 TMZ treated cases with unmethylated *MGMT* were used as the validation cohort. Clinicopathological information for these 147 cases is summarized in **Table 1**. There is no statistically significant difference for the clinicopathological features between the training and validation cohorts.

**Table 1:** Clinicopathological characteristics for *MGMT* unmethylated GBM patients who received TMZ.

		The 27K cohort		The 450K cohort		P-value
		Number	Percentage	Number	Percentage	
**Total**		107	100.00%	40	100.00%	
**Age**		20–89 (57)		23–78 (58)		0.99^a^
	< median	50	46.73%	19	47.50%	
	≥ median	57	53.27%	21	52.50%	
**Gender**						0.53^b^
	Male	69	64.49%	28	70.00%	
	Female	38	35.51%	12	30.00%	
**IDH**						0.32^b^
	Mutant	4	3.74%	0	0.00%	
	Wildtype	90	84.11%	37	92.50%	
	NA	13	12.15%	3	7.50%	
**TCGA defined subgroup**						0.10^b^
	Neural	10	9.35%	2	5.00%	
	Proneural	18	16.82%	6	15.00%	
	Classical	30	28.04%	12	30.00%	
	Mesenchymal	43	40.19%	12	30.00%	
	NA	6	5.61%	8	20.00%	
**Chr 7 gain/Chr 10 loss**						0.37^b^
	Combined alteration	73	68.22%	28	70.00%	
	No combined alteration	29	27.10%	12	30.00%	
	NA	5	4.67%	0	0.00%	

^a^P-value is calculated by the nonparametric test. ^b^P-value is calculated by the Chi-square tests.

### Analytical Approach

**Figure 1:**
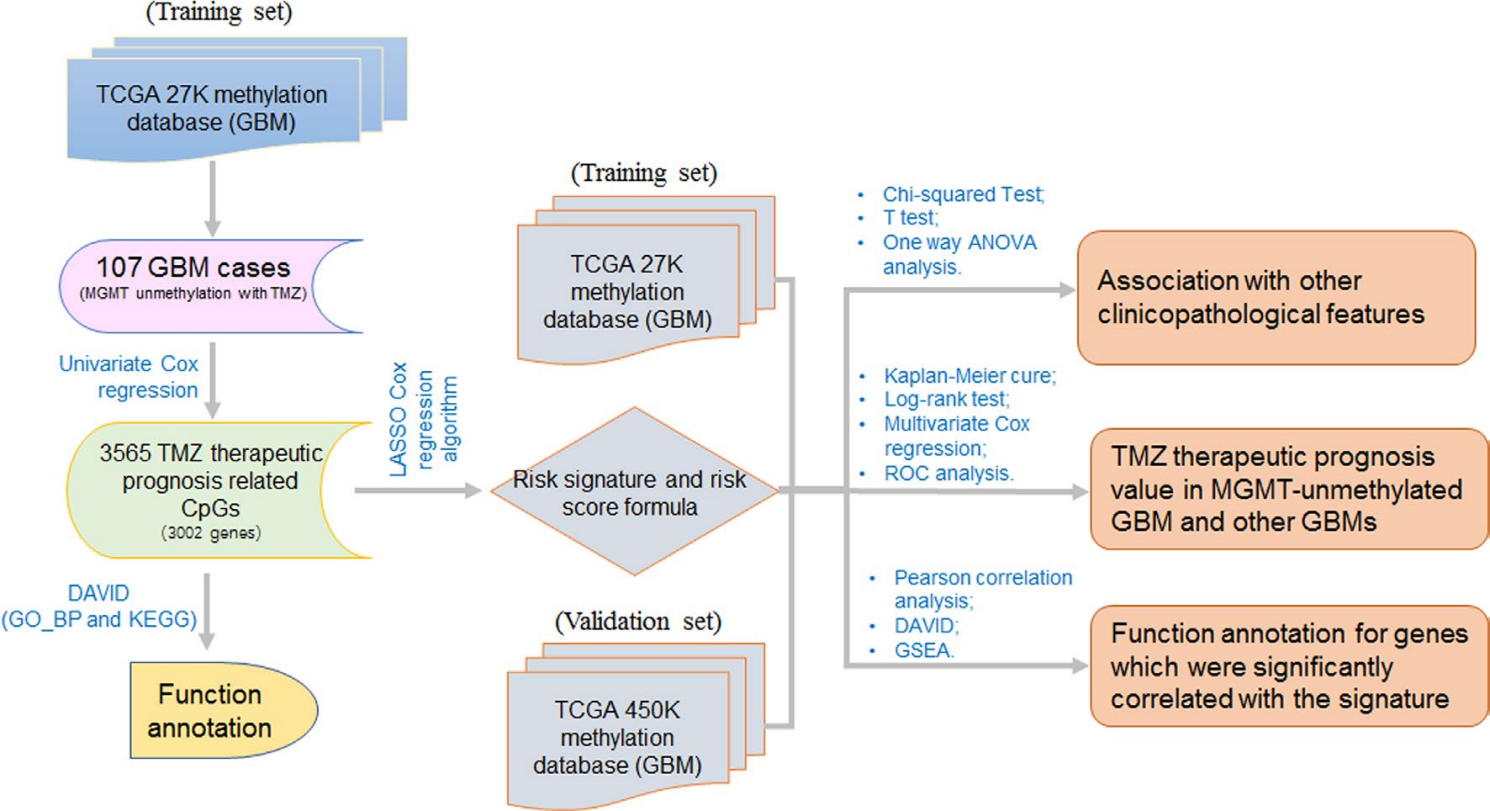
The workflow for this study. The workflow for the selection of TMZ therapeutic prognosis related CpGs, development and validation of a TMZ therapeutic prognostic risk signature, and the functional analysis of genes that are correlated with the signature risk score.

The approach and workflow for the selection of TMZ therapeutic prognosis associated CpGs, functional annotation for the genes corresponding to these CpGs, development and validation of a TMZ therapeutic prognostic risk signature, analysis of the correlation between the risk signature and other clinicopathological features, and the functional analysis of genes associated with the risk signature are summarized in **Figure 1**.

### Identification of the Risk Signature

We performed univariate Cox regression analysis of the CpGs methylation to identify CpGs significantly correlated with the prognosis of TMZ treated *MGMT* unmethylated GBM in the 27K cohort. Then, we used the least absolute shrinkage and selection operator (LASSO) Cox regression algorithm to develop an optimal risk signature with the minimum number of CpGs (Dai et al., 2018; Zhou et al., 2018; Chai et al., 2019d). Finally, a set of 31 CpGs and their coefficients were determined by the minimum criteria, which involves selecting the best penalty parameter λ associated with the smallest 10-fold cross validation within the training set. The risk score for the risk signature was calculated using the formula:

$$\text{Risk score }=\;\Sigma_{i=\;1}^{n}\;Coef_{i}\;*\;x_{i}$$

where *Coef* is the coefficient and *x_i_* is the beta-value of each selected CpGs. In both groups (cohorts), we used the beta-value [beta-value = the methylated signal/(methylated signal + unmethylated signal)] to represent the methylation level of each CpGs. Since the Risk score was calculated as a weighted sum of the methylation level of all selected CpG sites (Chai et al., 2019d; Chen et al., 2019a), we just used the original beta value of each CpG sites to calculate the risk scores.

We did not directly compare the samples in two different groups (cohorts). In order to avoid the bias caused by the different arrays, we only compared the methylation levels among samples in the same cohort. We first developed the risk signature in 107 samples used HumanMethylation27 (HM-27K) BeadChips. Then, we used another 40 samples to validate the prognostic value of the proposed signature. Patients were divided into “high-risk” and “low-risk” groups using the respective median risk score as the cutoff value in both the training and validation datasets.

### Bioinformatic Analysis

Significance analysis of microarray (SAM) was performed to identify differentially expressed genes within the low- and high- risk scores. We performed Gene Ontology (GO) and Kyoto Encyclopedia of Genes and Genomes (KEGG) pathway enrichment analyses with the Database for Annotation, Visualization, and Integrated Discovery (http://david.abcc.ncifcrf.gov/home.jsp) to functionally annotate genes corresponding to the CpGs with prognosis of TMZ treated MGMT unmethylated GBM and genes that were differentially expressed between the low- and high-risk groups in the 27K cohort. Gene Set Enrichment Analysis (GSEA) was performed to investigate the functions of genes that were differentially expressed between the low- and high-risk groups in the 27K cohort.

### Statistical Analysis

We used the nonparametric test to compare the distribution of age between the low- and high-risk groups, and Chi-square tests were used to compare the distribution of other clinicopathological features. A one-way analysis of variance was performed to compare the risk scores in patients grouped by the TCGA defined subtypes. Student’s *t* test was performed to compare the risk scores in patients grouped by other clinical or molecular-pathological characteristics.

Univariate and multivariate Cox regression analysis was performed to determine the prognostic value of the risk score and various clinical and molecular–pathological characteristics.

The Kaplan–Meier method with a two-sided log-rank test was used to compare the OS of patients stratified by the risk scores or other clinicopathological features. All statistical analyses were conducted using R v3.4.1 (https://www.r-project.org/), SPSS 16.0 (SPSS, Inc., Chicago, IL, USA) and Prism 7 (GraphPad Software, Inc., La Jolla, CA, USA).

## Results

### A Set of CpGs’ Methylation Profile Could Predict the TMZ Therapeutic Response of *MGMT* Unmethylated GBMs

**Figure 2:**
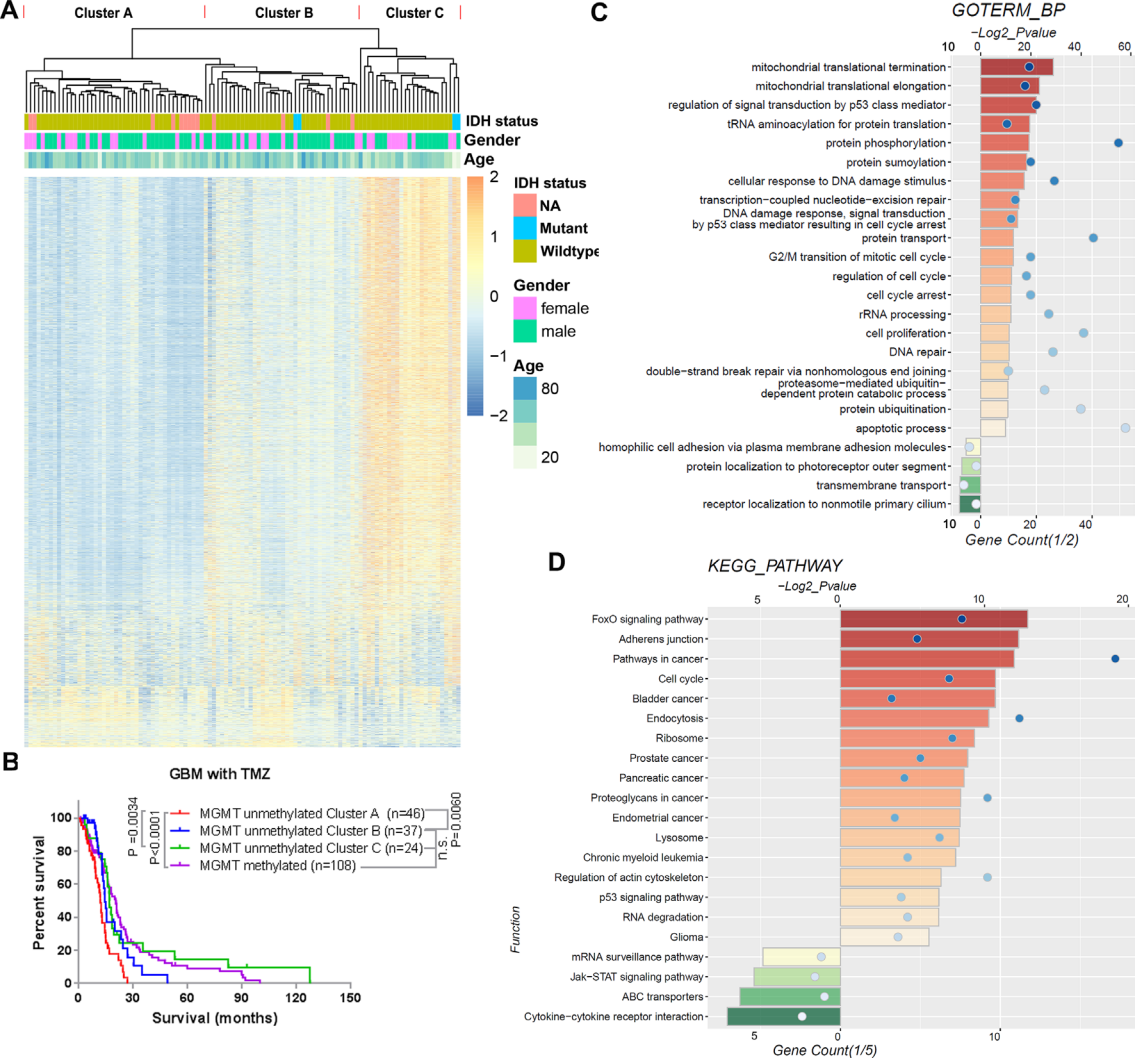
TMZ therapeutic prognosis associated CpGs’ methylation profile in *MGMT* unmethylated GBMs. **(A)** Heatmap showing the methylation levels of the 3,565 GpGs associated with the overall survival of TMZ treated patients with *MGMT* unmethylated GBMs. The *MGMT* unmethylated GBMs could be clustered into 3 clusters (Cluster A–C) according to the CpGs methylation levels. **(B)** Kaplan–Meier overall survival (OS) curves of TMZ treated *MGMT* unmethylated GBM patients (stratified by Cluster A–C) and TMZ treated *MGMT* methylated GBM patients. **(C**, **D)** GO biological process terms **(C)** and KEGG pathways **(D)** enriched among the genes positively and negatively corresponding to the 3,565 GpGs.

To assess the TMZ therapeutic response value of the methylation of CpGs, we performed univariate Cox regression analysis of all CpGs methylation levels in the 107 TMZ treated *MGMT* unmethylated GBMs of the 27K cohort. We found that the methylation levels of 3,565 CpGs were significantly correlated with the OS of these GBMs (**Supplementary Table 2**). Based on the methylation profile of these genes, we could divide the 107 TMZ treated *MGMT* unmethylated GBMs into 3 clusters (Cluster A–C) in the heatmap (**Figure 2A**). We observed that patients in the Cluster A had significantly shorter survival than patients in the Cluster B and C, and the patients in the Cluster B and C had a similar OS with the TMZ treated *MGMT* methylated GBM patients (**Figure 2B**).

We also investigated the functions of the respective genes for the 3,565 CpGs. Three thousand one hundred eighty-two of these CpGs methylation levels were found to have a HR < 1 and were considered protective-associated, and the remaining 383 CpGs methylation levels with a HR >1 were considered risk-associated. GO terms of biological progress (BP) and KEGG pathway analysis indicated that the genes corresponding to the protective-associated CpGs were enriched in the processes of mitochondrial translation, protein modification, cell cycle, DNA repair, others, and pathways in cancer (**Figures 2C, D**). In contrast, the genes corresponding to the risk-associated CpGs were mainly enriched in the cellular membrane-associated biological processes and pathways (**Figures 2C, D**).

### Identification of a 31-CpGs Panel as a TMZ Therapeutic Prognosis Risk Signature in *MGMT* Unmethylated GBMs

**Figure 3:**
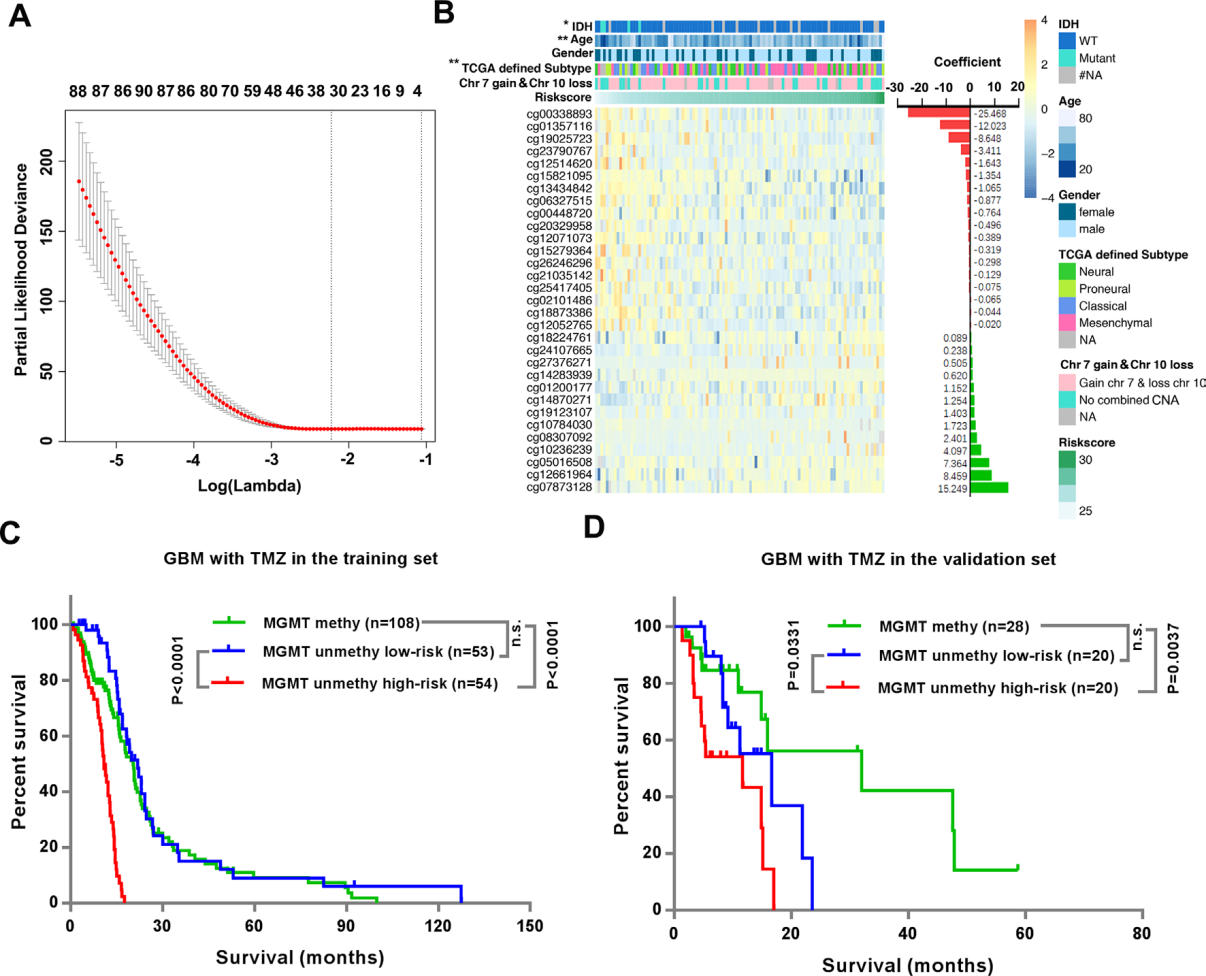
Identification of the risk signature could stratify the TMZ therapeutic prognosis of the MGMT unmethylated GBM. **(A)** Ten-fold cross validation for tuning parameter selection in the LASSO model. The minimum criterion was indicated by the dashed vertical line (left). **(B)** Heatmap shows the association of risk scores and clinicopathological features based on the methylation profile of the 31 CpGs in the signature. The coefficients were calculated by multivariate Cox regression analysis using LASSO. **(C–D)** Kaplan–Meier overall survival (OS) curves for TMZ treated patients with *MGMT* methylated GBMs, TMZ treated patients with *MGMT* unmethylated GBMs with low- or high-risk significance scores in the training set **(C)** and validation set **(D)**, respectively.

We next sought to develop a representative “risk signature” with a small number of CpGs to predict the TMZ therapeutic responses of the *MGMT* unmethylated GBMs. We applied the LASSO Cox regression algorithm to the 3,565 CpGs in 107 GBMs of the 27K cohort (**Figure 3A**). Finally, a total of 31 CpGs were contained in the risk signature, and the respective genes and the coefficients of these CpGs were also shown (**Figure 3B** and **Supplementary Table 3**). Twenty-four of the 31 CpGs are located in the CpG islands of prospective genes, and 5 of the other 7 CpGs are located within 200 bp of the transcription start site of the prospective genes (**Supplementary Table 3**). Most of the genes corresponding to the 31 CpGs have been reported to be involved in the tumorigenesis or prognosis of cancer, including *ATOH1, ATPIG1, ELL3, RBM15B, GATA4, TXN, DLX5, THSD4, Polr2d, LGALS3BP, HIST1H3D, FLRT1, IFI35* and *OSBPL5*. Among these genes, hypermethylation of *THSD4* has been reported to be associated with the prediction of prognosis in GBM (Ma et al., 2015), and *Polr2d* expression is associated with the therapy response of GBM (Serao et al., 2011).

We divided patients into high-risk and low-risk groups using their median risk-score as the cutoffs. We observed significant differences between the low- and high- risk groups with respect to *IDH* status (P = 0.0431), age (P = 0.0069) and TCGA defined subtype (0.0047), but no differences in gender or chromosome 7 gain combined with chromosome 10 loss (chr 7 gain and chr 10 loss) (**Figure 3B** and **Supplementary Table 4**).

Then we investigated the relationship between the risk signature and OS of TMZ treated *MGMT* unmethylated GBM patients. The data showed that patients with low-risk-scores had significantly longer OS than patients with high-risk-scores in both the training (P < 0.0001) and validation (P = 0.0331) datasets (**Figures 3C, D**). In addition, although the OS of *MGMT* methylated GBM patients was significantly longer than that of *MGMT* unmethylated GBM patients (**Supplementary Figure 1**), we noticed that the OS of *MGMT* unmethylated GBM patients in the low-risk group was similar to that of *MGMT* methylated GBM patients in both the training and validation datasets (**Figures 3C, D**).

### Association of the Risk Signature and Other Clinicopathological Features

Considering that the TMZ therapeutic prognosis value of the risk signature may be associated with other known clinicopathological features, we examined this in the *MGMT* unmethylated GBMs. We observed that the risk scores were only significantly different between patients stratified by age (P < 0.05), rather than gender, chr 7 gain and chr 10 loss, and the TCGA defined subtypes (**Supplementary Figure 2**). We did not compare the risk scores in patients with different *IDH* status, as there were only four *IDH*-mutant patients.

We also performed univariate and multivariate Cox regression analyses in the TMZ treated *MGMT* unmethylated GBMs of both the training and validation datasets. By univariate analysis, the risk score [hazard ratio (HR) = 12.674 (7.661–20.968) in the training set; HR = 1.685 (1.058–2.682) in the validation set] and age [HR = 1.029 (1.009–1.048) in the training set; HR = 1.075 (1.023–1.13) in the validation set] were significantly correlated with the OS in both two datasets (**Table 2**). When including these factors into the multivariate Cox regression analysis, the risk score remained significantly associated with the OS in the training [HR = 12.748 (7.767–21.173)] and validation [HR = 2.157 (1.139–4.086)] datasets (**Table 2**). These results indicated that the risk score can independently predict the TMZ therapeutic prognosis of patients with *MGMT* unmethylated GBMs.

**Table 2:** Univariate and multivariate Cox regression analyses for the risk score in the training and validation set, respectively.

		Univariate Cox regression				Multivariate Cox regression			
		P-value^a^	HR	95% CI		P-Value^b^	HR	95% CI	
				Lower	Higher			Lower	Higher
The training set	Age	**0.028**	1.029	1.009	1.048	0.858	0.998	0.974	1.022
	Gender	0.15	0.705	0.438	1.134	–	–	–	–
	Risk score	**<0.001**	12.674	7.661	20.968	**<0.001**	12.748	7.676	21.173
									
The validation set	Age	**0.004**	1.075	1.023	1.13	**0.03**	1.093	1.03	1.16
	Gender	0.654	0.802	0.305	2.107	–	–	–	–
	Risk score	**0.028**	1.685	1.058	2.682	**0.018**	2.157	1.139	4.086

^a^The P-value is the sig. value in the univariate cox regression, and the method is Enter; ^b^The p-value is the sig. value in the multivariate cox regression analysis, and the method is Enter. P-value <0.05 are highlighted by bold front.

We also investigated the association of risk scores and clinicopathological features in all GBM. We found that the risk scores were only significantly different between patients with different *IDH* status (P < 0.0001) or between Proneural subtype and Mesenchymal subtype (P < 0.01), but not between patients stratified by age, gender, *MGMT* promoter methylation status, chr 7 gain and chr 10 loss, or treated with or without TMZ (**Supplementary Figure 3**).

### Prognosis Value of the Risk Signature in Stratified GBMs

**Figure 4:**
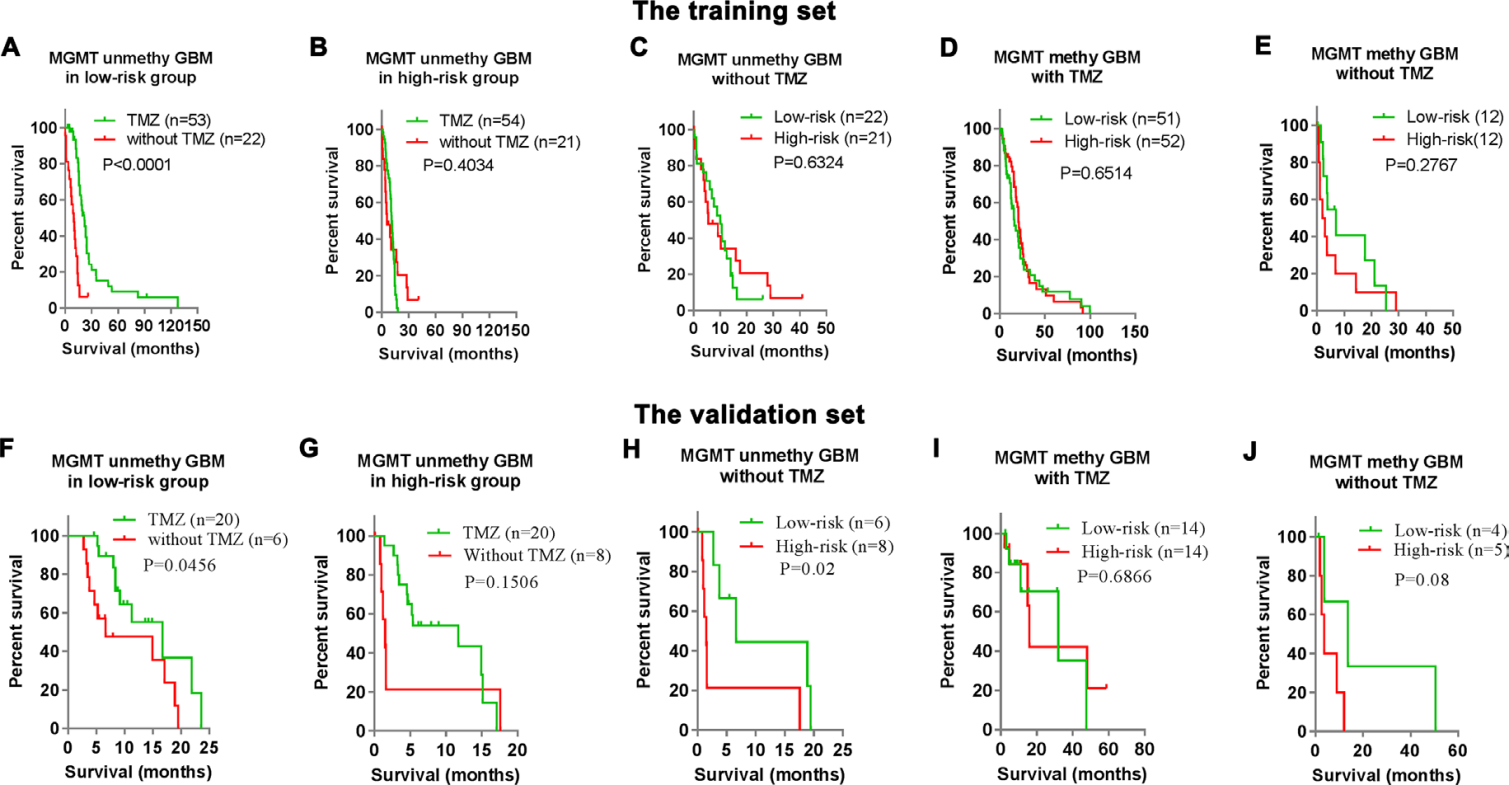
Clinical outcomes prediction of the signature in patients with stratified GBMs. **(A**–**B)** Kaplan–Meier overall survival (OS) curves for *MGMT* unmethylated GBM patients with or without TMZ treatment in the low-risk group **(A)** and high-risk groups **(B)** of the training set. **(C**–**E)** Kaplan–Meier overall survival (OS) curves for stratified GBM patients **(C)** *MGMT* unmethylated GBM without TMZ; **(D)** *MGMT* methylated GBM with TMZ; **(E)** *MGMT* methylated GBM without TMZ) with low- or high-risk scores in the training set. **(F**–**J)** Kaplan–Meier overall survival (OS) curves for stratified GBM patients in the validation set.

To further understand the TMZ therapeutic prognostic value of the risk signature in *MGMT* unmethylated GBMs, we compared the OS of *MGMT* unmethylated GBMs patients stratified by TMZ treatment status in the low-risk and high-risk groups respectively. The results indicated that patients with TMZ treatment had longer OS than that of patients without TMZ treatment in the low-risk group of both the training set (P < 0.0001, **Figure 4A**) and the validation set (P = 0.0456, **Figure 4F**). In contrast, there was no significant difference between patients with or without TMZ treatment in the high-risk group (**Figures 4B, G**).

We also investigated the prognostic value of the risk signature in other stratified GBMs. We respectively stratify the GBM patients into four subgroups according to *MGMT* status and TMZ treatment option. In the training set, the risk signature could not stratify the prognosis of three subgroups (TMZ non-treated MGMT unmethylated GBM, TMZ treated MGMT methylated GBM, and TMZ non-treated MGMT methylated GBM) (**Figures 4C–E**). Similar results could also be observed in the validation set except TMZ non-treated *MGMT* unmethylated GBM (**Figures 4G, H**).

### The Potential Functions Underlying the TMZ Therapeutic Prognostic Value of the Risk Signature

**Figure 5:**
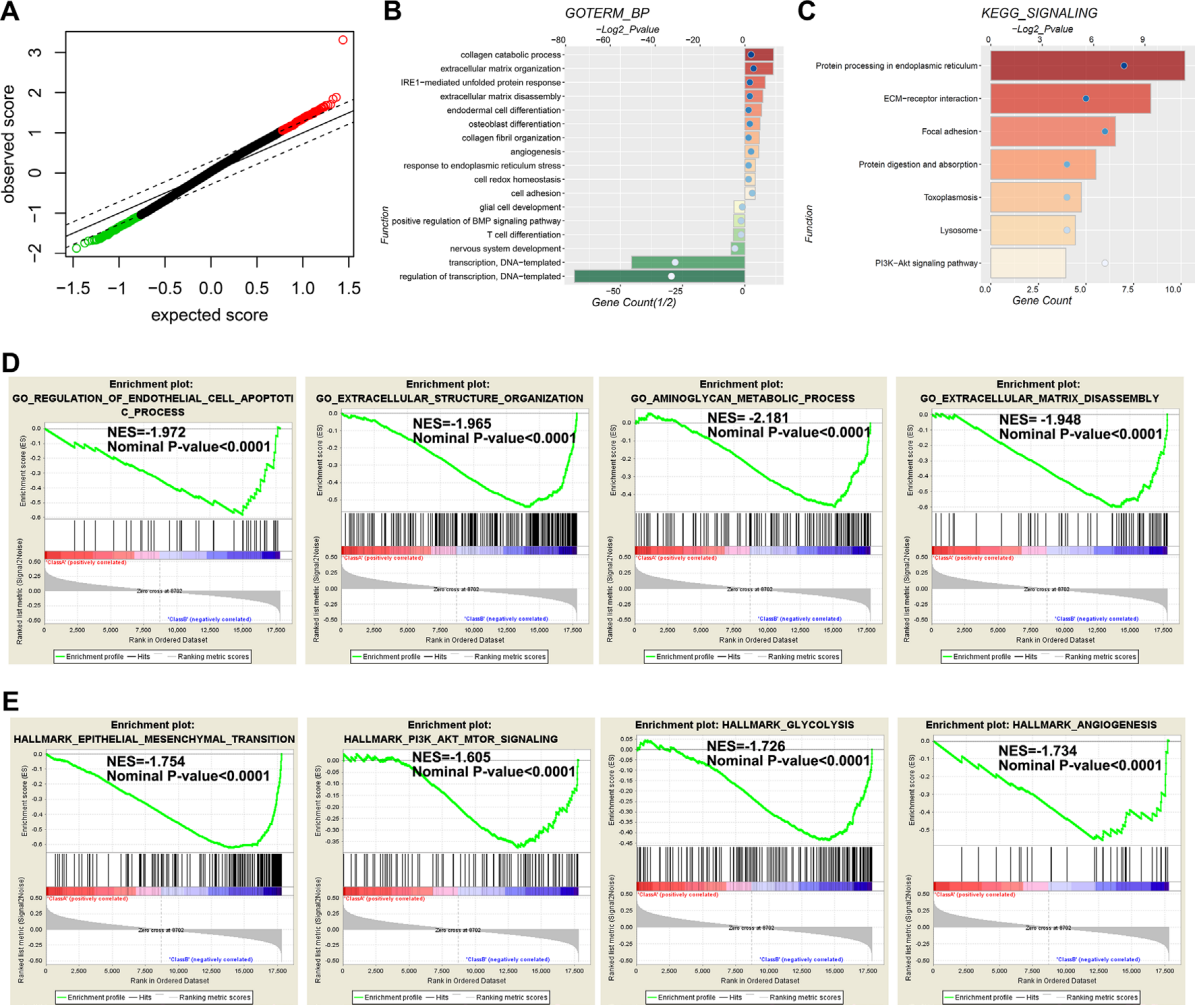
Functional annotation for genes differentially expressed between low- and high-risk groups. **(A)** The differential genes between low- and high-risk groups are shown by green (enriched in the low-risk group) and red (enriched in the high-risk group) dots. **(B**–**C)** Go analysis **(B)** and KEGG analysis **(C)** are used to evaluate differential genes between low-and high-risk groups. **(D** and **E)** GSEA analysis reveals the biological processes **(D)** and cancer hallmarks **(E)** enriched in the high-risk groups.

To determine the functional differences between the high-risk and low-risk cases of the TMZ treated *MGMT* unmethylated GBM in the 27K cohort, we identified the differentially (P < 0.05) expressed genes by SAM (**Figure 5A**). GO and KEGG analyses revealed that extracellular matrix related biological processes and signaling pathways were significantly enriched in the high-risk group (**Figures 5B, C**). In contrast, the biological processes of T cell differentiation, nervous system development, and transcription were significantly enriched in the low-risk group (**Figure 5B**). Meanwhile, GSEA also indicated that the high-risk cases showed enrichment of “regulation of endothelial cell apoptotic process,” “extracellular structure organization,” “aminoglycan metabolic process,” and “extracellular matrix disassembly biological progresses” (**Figure 5D**). Moreover, the hallmarks of “epithelial–mesenchymal transition,” “PI3K-AKT-mTOR signaling,” “glycolysis”, and “angiogenesis” also enriched in the high-risk cases (**Figure 5E**). The results indicated that the extracellular matrix related functions and mesenchymal phenotype could contribute to the TMZ-resistant of glioma.

## Discussion

Undoubtedly, *MGMT* promoter methylation status is critical for the chemotherapeutic management of glioma, especially for GBM (Hegi et al., 2005; Chai et al., 2019a; Chai et al., 2019e). However, whether TMZ should be withheld from patients with GBMs that lack *MGMT* promoter methylation is still under debate, and some of these patients indeed benefit from the treatment (Wick et al., 2014). Thus, it is critical to uncover novel biomarkers to identify TMZ-sensitive individuals with *MGMT* promoter unmethylated GBMs. In this study, we successfully developed a 31-CpG methylation signature which could identify the TMZ-sensitive GBMs in the *MGMT* promoter unmethylated GBMs from both the training and validation datasets, and OS of these TMZ-sensitive GBMs is similar to that of the *MGMT* promoter methylated GBMs after TMZ treatment in both two datasets. Considering the robust and reproducible nature of DNA methylation in the classification of brain tumors, this signature has great value in predicting the TMZ sensitivity of the GBMs that lack *MGMT* promoter methylation.

In this study, we systematically investigated 107 *MGMT* promoter unmethylated GBMs to obtain the TMZ therapeutic prognostic value of each of the CpGs that were included in the HM-27K BeadChip, and we identified that 3,565 CpGs are significantly associated with the OS of these GBMs. Previous studies have indicated that abnormal metabolism could alter the response of tumor cells to chemotherapy through inhibiting the activities of DNA repair enzymes (Gusyatiner and Hegi, 2018). DNA instability and DNA injury repair have been linked to the chemo-resistance of cancer cells (Kanai et al., 2012; Roos et al., 2018; Zhao et al., 2018a; Ha Thi et al., 2019; Zhang et al., 2019). Here we also investigated the functions of genes corresponding to the 3,565 CpGs, and the results indicated that biological processes or pathways of mitochondrial translation, cell cycle and DNA repair could be involved in the TMZ-sensitivity of *MGMT* promoter unmethylated GBMs. Given that DNA proliferation rate is positively correlated to the sensitivity to chemotherapy (Li et al., 2017; Krell et al., 2019; Qiang et al., 2018), our finding supports that transcriptional activities of genes enriched in mitochondria, DNA injury and repair, and cell cycle processes could be important in the sensitivity of GBM cells to TMZ chemotherapy.

The extracellular matrix (ECM) components and their partners, including the glycosaminoglycans, glycoproteins, and proteoglycans, play a crucial role in the glioma invasion through promoting tumor cell migration and angiogenesis (Ferrer et al., 2018). The up-regulation of ECM partners, such as CD44, has been acknowledged as a marker for the “proneural–mesenchymal transition” of GBM cells (Yang et al., 2017). Here, we noticed that not only the hallmark of epithelial–mesenchymal transition but also ECM related biological processes and pathways were highly enriched in the *MGMT* unmethylated GBMs with the high-risk score, indicating that enhanced ECM activities could be involved in the TMZ-resistance of GBMs. This may be associated with the roles of ECM in regulating the extracellular microenvironments and intracellular signaling pathways (Wang et al., 2018). Chemokine (C-X-C motif) ligand 12 (CXCL12) and its receptors CXCR4 and CXCR7, which are stored in or attached to the ECM, are extremely important in forming a more invasive and resistant phenotype of glioma (Gatti et al., 2013; Zhao et al., 2018a). Recently, we also identified that the glycoprotein ADAMTS4, which is important for the upregulation of integrins, is also a novel immune-related biomarker for the primary GBM (Zhao et al., 2019). Transforming growth factor-beta (TGF-β), an ECM-bound bioactive factor, is involved in both the activation of NF-κB signaling and mesenchymal transition of GBM (Song et al., 2018; Batlle and Massague, 2019). Both of these two processes have been involved in the TMZ-resistance of GBM (Ming et al., 2017; Yang et al., 2017; Chai et al., 2019c; Chai et al., 2019d). All of these emphasize the value of the ECM in glioma TMZ sensitivity. Thus, the ECM and microenvironment should not be neglected in drug development, especially in developing an ideal *in vitro* drug screening model for glioma.

Chr 7 gain and Chr 10 loss is quite common in GBM (Bady et al., 2016; Chai et al., 2019a). Patients with high-grade gliomas harboring deletions of chromosomes 9p and 10q may benefit more from TMZ treatment (Wemmert et al., 2005), and the MGMT resides on chromosome 10q. Here, we also investigated the association between the risk signature and deletion of one copy of chromosome 10, and the results indicated that the predictive value of the risk signature was not affected by the status of Chr 7 gain and Chr 10 loss. This finding excludes the possibility that the predictive value of the risk signature may be caused by the unbalanced *MGMT* expression between GBM with or without Chr 7 gain and Chr 10 loss. Moreover, we have reported that chromosome 10/10q deletion does not significantly affect *MGMT* expression of GBM in the TCGA dataset (Chai et al., 2019a).

In conclusion, our findings reveal the predictive value of DNA methylation profiling in GBMs with an unmethylated *MGMT* promoter. The developed 31-CpG methylation signature could accurately predict the TMZ-sensitivity of *MGMT* promoter unmethylated GBMs. Though the risk signature still needs to be confirmed in future prospective studies with specific test kits, our current findings can promote our understanding of the roles of DNA methylation in GBMs with an unmethylated *MGMT* promoter and also offer a very promising TMZ-sensitivity predictive signature for these GBMs.

## Data Availability Statement

All methylation and clinical data used in this study were available from the TCGA database (http://cancergenome.nih.gov). Other information is available through contacting the corresponding authors.

## Author Contributions

R-CC conceived and designed the study, R-CC, Y-ZC, and Q-WW crafted the literature search, figures, and tables and were responsible for the writing and critical reading of the manuscript. K-NZ, J-JL, and HH contributed to the data analysis and the critical reading of the manuscript. FW, Y-QL, and Y-ZW supervised the analysis and contributed to the critical reading of the manuscript.

## Funding

This work was supported by the National Key Research and Development Program of China (2018YFC0115604), the National Natural Science Foundation of China (81773208, 81802994), the National Natural Science Foundation of China (NSFC)/Research Grants Council (RGC) Joint Research Scheme (81761168038), Beijing Municipal Administration of Hospitals’ Mission Plan (SML20180501, 2018.03-2022.02).

## Conflict of Interest

The authors declare that the research was conducted in the absence of any commercial or financial relationships that could be construed as a potential conflict of interest.
