# Supplementary material for: A Novel DNA Methylation-Based Signature Can Predict the Responses of MGMT Promoter Unmethylated Glioblastomas to Temozolomide
Source: Front Genet. 2019 Sep 27;10:910. doi: 10.3389/fgene.2019.00910 (PMC6776832; doi:10.3389/fgene.2019.00910)
Supplement: Supplementary file 6 [file DataSheet_1.pdf]

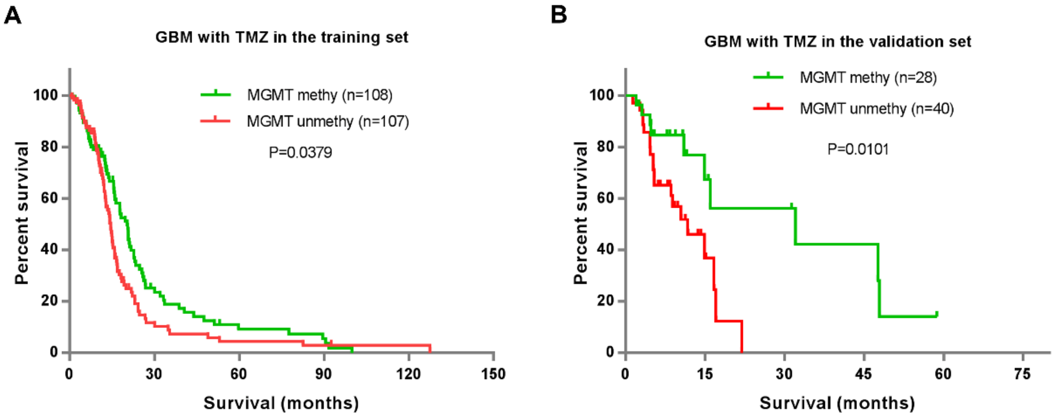

Figure S1. The overall survival for TMZ treated patients with *MGMT* methylated or un-methylated GBM.

A-B. Kaplan–Meier survival curves for TMZ treated GBM patients stratified by the MGMT methylation status in the training set (A) and the validation set (B), respectively.

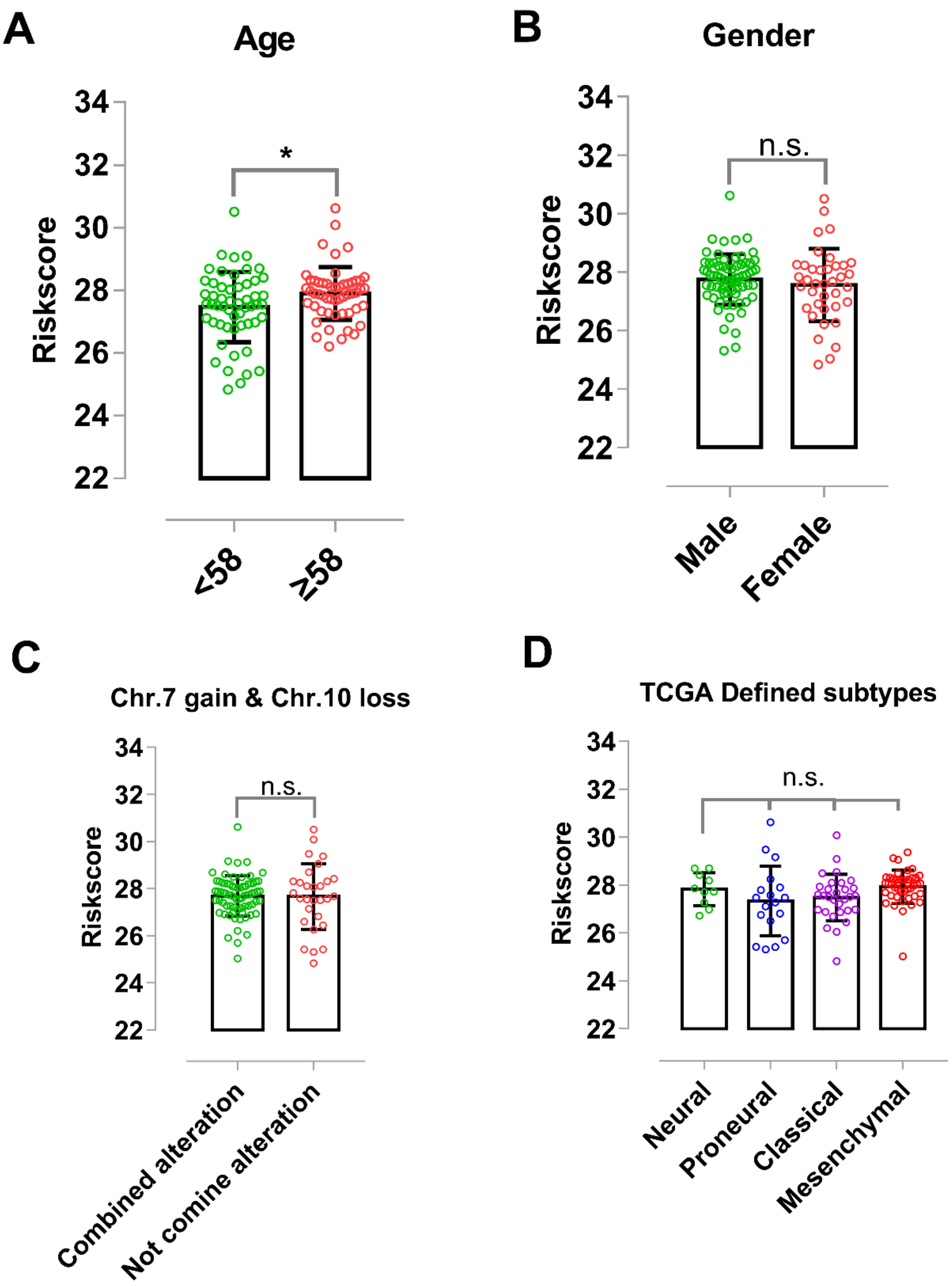

Figure S2. Relationship between the risk score and clinicopathological features of TMZ treated *MGMT* un-methylated GBM.

A-D Distribution of risk scores in the TMZ treated MGMT unmethylated GBM stratified by Age (A), Gender (B), Chromosome 7 gain and Chromosome 10 loss status (C), and TCGA defined subtypes (D).

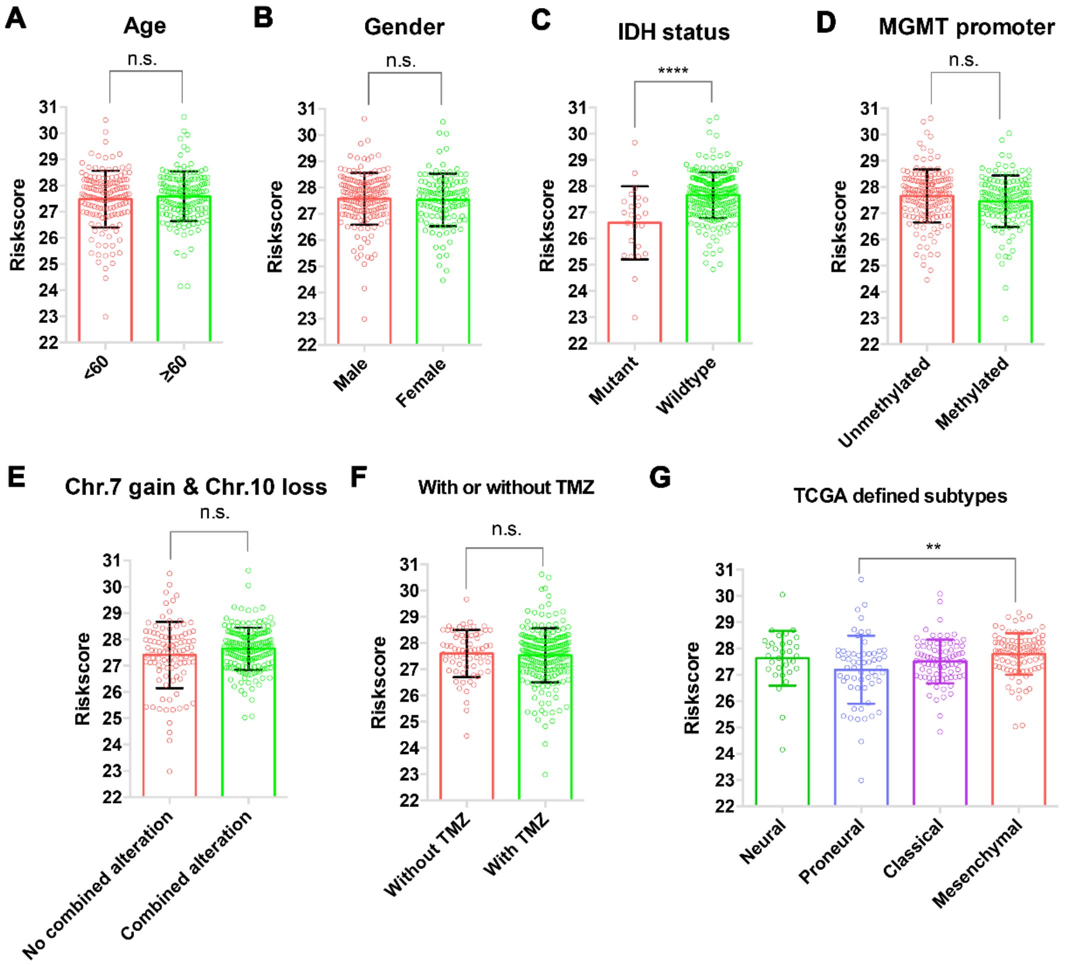

Figure S3. Relationship between the risk score and the clinicopathological features of all GBM. A-D Distribution of risk scores in the GBM by Age (A), Gender (B), IDH status (C), MGMT promoter methylation status (D), Chromosome 7 gain and Chromosome 10 loss status (E), with or without TMZ (F), and TCGA defined subtypes (G).
